# The association between area-level residential instability and gray matter volume changes

**DOI:** 10.1192/j.eurpsy.2022.2033

**Published:** 2022-09-01

**Authors:** B. Ku, J. Addington, C. Bearden, K. Cadenhead, T. Cannon, M. Compton, B. Cornblatt, B. Druss, M. Keshavan, D. Mathalon, T. Mcglashan, D. Perkins, L. Seidman, W. Stone, M. Tsuang, S. Woods, E. Walker

**Affiliations:** 1 Emory, Psychiatry And Behavioral Sciences, Atlanta, United States of America; 2 University of Calgary, Psychiatry, Calgary, Canada; 3 UCLA, Psychiatry, Los Angeles, United States of America; 4 UCSD, Psychiatry, San Diego, United States of America; 5 Yale, Psychiatry, New Haven, United States of America; 6 Columbia, Psychiatry, New York, United States of America; 7 Zucker Hillside Hospital, Psychiatry, Long Island, United States of America; 8 Emory, Department Of Health Policy And Management, Atlanta, United States of America; 9 Harvard, Psychiatry, Boston, United States of America; 10 UCSF, Psychiatry, San Francisco, United States of America; 11 University of North Carolina, Psychiatry, Chapel Hill, United States of America; 12 Emory, Psychology, Atlanta, United States of America

**Keywords:** clinical high risk for psychosis, grey matter volume, residential instability, area-level factors

## Abstract

**Introduction:**

Area-level residential instability (ARI), an index of social fragmentation, has been shown to explain the association between urbanicity and psychosis. Urban upbringing has been shown to be associated with decreased gray matter volumes (GMV)s of brain regions corresponding to the right caudal middle frontal gyrus (CMFG) and rostral anterior cingulate cortex (rACC).

**Objectives:**

We hypothesize that greater ARI will be associated with reduced right posterior CMFG and rACC GMVs.

**Methods:**

Data were collected at baseline as part of the North American Prodrome Longitudinal Study. Counties where participants resided during childhood were geographically coded using the US Censuses to area-level factors. ARI was defined as the percentage of residents living in a different house five years ago. Generalized linear mixed models tested associations between ARI and GMVs.

**Results:**

This study included 29 HC and 64 CHR-P individuals who were aged 12 to 24 years, had remained in their baseline residential area, and had magnetic resonance imaging scans. ARI was associated with reduced right CMFG (adjusted β = -0.258; 95% CI = -0.502 – -0.015) and right rACC volumes (adjusted β = -0.318; 95% CI = -0.612 – -0.023). The interaction terms (ARI X diagnostic group) in the prediction of both brain regions were not significant, indicating that the relationships between ARI and regional brain volumes held for both CHR-P and HCs.

**Conclusions:**

Like urban upbringing, ARI may be an important social environmental characteristic that adversely impacts brain regions related to schizophrenia.

**Disclosure:**

No significant relationships.

